# HIV-1 Nucleocapsid Protein Binds Double-Stranded DNA in Multiple Modes to Regulate Compaction and Capsid Uncoating

**DOI:** 10.3390/v14020235

**Published:** 2022-01-25

**Authors:** Helena Gien, Michael Morse, Micah J. McCauley, Jonathan P. Kitzrow, Karin Musier-Forsyth, Robert J. Gorelick, Ioulia Rouzina, Mark C. Williams

**Affiliations:** 1 Department of Physics, Northeastern University, Boston, MA 02115, USA; h.gien@northeastern.edu (H.G.); mi.morse@northeastern.edu (M.M.); m.mccauley@northeastern.edu (M.J.M.); 2 Department of Chemistry and Biochemistry, Center for Retroviral Research and Center for RNA Biology, Ohio State University, Columbus, OH 43210, USA; kitzrojp@gmail.com (J.P.K.); musier@chemistry.ohio-state.edu (K.M.-F.); irouzina@gmail.com (I.R.); 3 AIDS and Cancer Virus Program, Leidos Biomedical Research, Inc., Frederick National Laboratory for Cancer Research, Frederick, MD 21702, USA; gorelicr@mail.nih.gov

**Keywords:** DNA condensation, optical tweezers, atomic force microscopy, capsid uncoating, HIV-1 nucleocapsid protein

## Abstract

The HIV-1 nucleocapsid protein (NC) is a multi-functional protein necessary for viral replication. Recent studies have demonstrated reverse transcription occurs inside the fully intact viral capsid and that the timing of reverse transcription and uncoating are correlated. How a nearly 10 kbp viral DNA genome is stably contained within a narrow capsid with diameter similar to the persistence length of double-stranded (ds) DNA, and the role of NC in this process, are not well understood. In this study, we use optical tweezers, fluorescence imaging, and atomic force microscopy to observe NC binding a single long DNA substrate in multiple modes. We find that NC binds and saturates the DNA substrate in a non-specific binding mode that triggers uniform DNA self-attraction, condensing the DNA into a tight globule at a constant force up to 10 pN. When NC is removed from solution, the globule dissipates over time, but specifically-bound NC maintains long-range DNA looping that is less compact but highly stable. Both binding modes are additionally observed using AFM imaging. These results suggest multiple binding modes of NC compact DNA into a conformation compatible with reverse transcription, regulating the genomic pressure on the capsid and preventing premature uncoating.

## 1. Introduction

**Figure 1:**
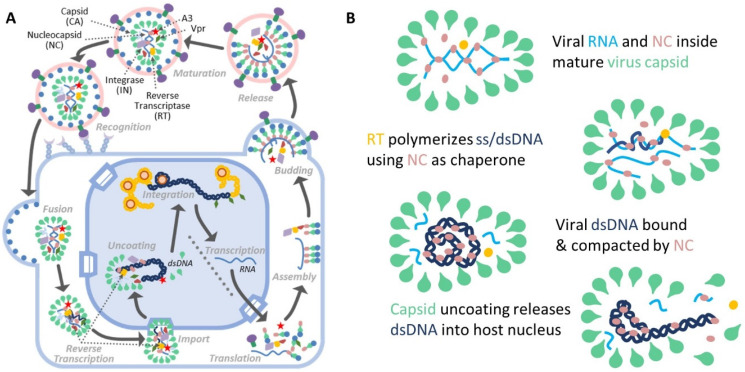
Overview of the role of NC in HIV replication. (**A**) NC is critical to multiple steps of the full lifecycle of HIV. As a subdomain of the Gag protein, NC is critical for packaging vRNA in the viral particle. When the viral particle matures, NC is cleaved from Gag and is contained inside the capsid with the vRNA. NC acts as a chaperone for reverse transcription of the viral RNA into DNA and binds and aggregates nucleic acids with high affinity. (**B**) NC binds and condenses viral DNA during the reverse transcription. NC acts as a chaperone during the synthesis of (−)DNA using vRNA as a template followed by synthesis of the complementary (+)DNA. For the full ~10 kbp viral DNA to be contained inside the capsid, which has a diameter comparable in length to the natural persistence length of DNA, the DNA must be compacted to a volume much smaller than predicted by the natural thermodynamic fluctuations of uncondensed DNA. Once the capsid uncoats, the viral DNA and viral proteins will be released into the host cell nucleus, enabling integration.

HIV-1 nucleocapsid protein (NC) is essential to viral replication. NC is initially expressed as a subdomain of the HIV-1 polyprotein Gag [1], the primary structural protein of HIV-1 that is solely and strictly necessary for viral particle formation. The NC domain of Gag and its binding affinity to nucleic acids is essential for the assembly of immature viral particles (Figure 1). As the viral particle matures, Gag is cleaved multiple times, resulting in the release of mature viral proteins including NC (sometimes referred to as NCp7), which binds to and condenses the viral RNA (vRNA) inside the mature capsid. NC also acts as a chaperone during reverse transcription (RT) [2], in which single-stranded (ss) minus-strand DNA ((−)DNA), complementary to the packaged vRNA, is first synthesized. After degradation of the vRNA into 8–17 nucleotide (nt) long oligonucleotides by the RNaseH activity of reverse transcriptase, double-stranded (ds) plus-strand DNA ((+)DNA) is synthesized and the remaining vRNA dissociates from the (−)DNA template, resulting in the full length proviral dsDNA. The mature HIV-1 viral capsid is roughly conical in shape, measuring approximately 100 nm along its long axis and 50 nm in diameter. The capsid contains the viral genome (two vRNA molecules), viral proteins (including NC, reverse transcriptase, integrase, Vpr, among others), and some host proteins, such as APOBEC3 proteins. While early experiments suggested that the capsid disassembles soon after entry into the host cell [1], more recent evidence points to successful viral infection associated with later capsid uncoating after the completion of RT [3,4,5,6,7,8,9,10,11,12,13,14,15,16,17,18,19,20]. Moreover, the intact capsid was found to be transported through the nuclear pore into the nucleus, where RT is completed, followed by uncoating about two hours afterwards in the vicinity of the proviral DNA integration site [3,4,5]. Importantly, RT stalling by RT inhibitors was observed to strongly delay capsid uncoating [3,6,8,11]. While the relative progression of RT and capsid uncoating has been established, the mechanics and timing of uncoating remain enigmatic. It has been proposed theoretically [21] and observed during RT in the purified capsid [6,8] that the synthesis of full length proviral DNA can induce sufficient pressure on the capsid to cause forcible disassembly (i.e., uncoating). Fully understanding the relationship between the progression of RT, internal DNA pressure, capsid deformation, and capsid uncoating requires knowledge of the NC-induced conformation of the rigid proviral dsDNA within the viral core. While interactions of ssRNA and ssDNA with HIV-1 NC have been studied extensively, complexes of DNA and NC remain less well understood [22,23,24].

In this study, we directly measure NC binding and compaction of single long dsDNA substrates (7.2, 8.1, and 48.5 kbp) isolated and manipulated by an optical tweezers system. We observe the real-time kinetics associated with NC binding to, dissociating from, and reorganizing the DNA substrate, the forces bound proteins exert on the substrate, and the geometry of the fully compacted protein-dsDNA complex. We observe that NC-induced DNA compaction has many features in common with DNA condensation induced by multivalent cations, though much stronger in effect [25]. We also image compacted NC-dsDNA complexes using atomic force microscopy (AFM). NC exhibits similar condensation activity on all three DNA substrates/sequences tested (genomic dsDNA from bacteriophages lambda and M13 and baculovirus plasmid pBACgus11), demonstrating the universality and sequence independence of NC-mediated DNA condensation. The in vitro characterization of the modes of NC-DNA binding and compaction reported here are likely critical to the HIV-1 lifecycle and allow us to propose new hypotheses that can be further tested in both in vitro assays and fully mature viral particles.

## 2. Materials and Methods

### 2.1. Protein Purification and DNA Substrate

Recombinant wild-type NL4-3 HIV-1 NCp7, 55 amino acid protein, without fluorescent label was produced and purified as described previously [26,27]. For fluorescent labeling, NCp7 was also produced and purified using a plasmid encoding an NC variant with an additional cysteine (A54C). A54C NCp7 (200 µg) was resuspended in 200 µL of labeling buffer (25 mM Tris-HCl [pH 7.5], 100 µm TCEP, 25 µM ZnCl_2_, 500 mM NaCl_2_) and buffer-exchanged at least three times using labeling buffer and Amicon^®^ Ultra 0.5 mL 3000 molecular weight cut-off centrifugal filters to ensure chelation of zinc ions to the NCp7 zinc fingers. The NCp7 A54C concentration after buffer exchange was measured via UV-Vis (ε_280_ = 5500 M^−1^cm^−1^). Two molar equivalents of Alexa 488-maleimide (dissolved in DMSO) was added to the protein suspension and incubated at room temp for 30 min. The labeling reaction was then quenched using 20 molar equivalents of β-mercatoethanol. Labeled protein was dialyzed overnight into labeling buffer to remove free dye. Labeled protein concentration and labeling efficiency (~30%) was determined via UV-Vis (NCp7 A54C: ε_280_ = 5500 M^−1^cm^−1^; Alexa 488: ε_495_ = 73,000). The fluorescent labeling efficiency of the protein was kept intentionally low to limit off-target labeling of the cysteine residues located within the two zinc fingers. Quantifying results from labeling reactions using NC with and without the A54C point mutation confirmed that off-target labeling was low (~5%, see Figure S1).

DNA substrates for optical tweezers experiments were prepared by biotinylating both ends of linearized baculovirus transfer plasmid pBACgus11 (gift from Borja Ibarra, IMDEA Nanociencia) (8.1 kbp) or lambda DNA (Roche, Basel, Switzerland) (48.5 kbp). For pBACgus11, the plasmid was double digested by BamHI and SacI (New England Biolabs) then incubated with a 10:1 excess of biotinylated oligonucleotides complementary to the resulting overhangs (sequences in Table S1). The annealed oligonucleotides were ligated using T4 DNA ligase (New England Biolabs, Ipswich, MA, USA) and free oligonucleotides were removed using gel purification, as previously described [28,29]. The ends of the lambda DNA were biotinylated in the same manner, with biotinylated oligonucleotides complementary to the DNA overhangs ligated to both ends [30]. DNA substrates for AFM imaging were prepared by linearizing M13mp18 DNA (New England Biolabs) with BamHI.

### 2.2. Optical Tweezers

Experiments with unlabeled proteins were performed on a custom-built optical tweezers system [28,29,31]. Biotinylated DNA was tethered between two streptavidin-coated beads. One bead was held by a glass micropipette tip moved by a piezo electric stage and one bead is held by a stationary counterpropagating dual-beam laser trap. The position of the piezo stage controls DNA extension and the deflection of the trapping laser is measured to determine DNA tension. Simultaneously, bright field images of both beads were taken to determine absolute DNA extension and correct for drift. The sample chamber is connected to multiple inlet tubes, which are used to exchange the buffer around the trapped DNA molecule. All experiments were done with ≥3 replicates with different DNA molecules and error bars in plots represent standard error unless otherwise noted.

Experiments with fluorescently-labeled proteins were performed on a C-Trap combined optical tweezers and fluorescent imaging system (LUMICKS, Amsterdam, Netherlands). DNA was tethered as described above, but with both beads held by movable laser traps. The sample chamber contains five laminar flow channels, allowing captured DNA molecules to be physically moved into different buffers. Confocal fluorescent images were obtained using a scanning laser at peak wavelength 488 nm for imaging NC labeled with Alexa 488.

### 2.3. AFM Imaging

Linearized M13mp18 dsDNA was diluted to a concentration of 50 pM (~200 pg/μL) and incubated with varying concentrations of NC for 1 min in a buffer containing 25 mM NaCl, 10 mM MgCl_2_, 10 mM Hepes, pH 7.5. Sample (20 μL) was deposited on a freshly cleaved mica surface pretreated with a 100 mM NiCl_2_ solution [32]. The sample was then washed with a buffer containing 25 mM NaCl, 10 mM NiCl_2_, 10 mM Hepes, pH 7.5. Samples were imaged with a MultiMode 8 AFM and Nanoscope V controller (Bruker) using peak force tapping mode and analyzed using Gwyddion software (version 2.55).

## 3. Results

### 3.1. Incubation of a Single Long DNA Molecule with Fluorescently-Labeled NC Reveals a Bright, Condensed Globule

**Figure 2:**
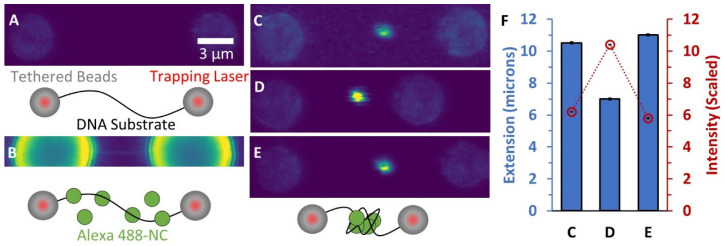
Binding of fluorescently-labeled NC to DNA. (**A**) Confocal scanning laser images (40 nm pixel size) and descriptive cartoons show two beads held by laser traps with an unlabeled DNA substrate tethered in between. The beads show weak autofluorescence and the DNA is not visible. (**B**) Alexa 488-NC is flowed into the sample chamber and binds the DNA (image magnification and brightness are enhanced to show faint line associated with NC binding uniformly along the stretched DNA). (**C**) When the DNA extension is decreased by bringing the two beads closer together, a large cluster of labeled NC forms along the substrate. The cluster becomes brighter when the substrate extension is further decreased (**D**) but becomes fainter again when extension is increased (**E**). (**F**) Quantification of the globule fluorescence intensity in each image compared to the substrate extension indicates that more labeled NC is incorporated into the globule as the end-to-end extension of the DNA is decreased. Error bars represent propagated error based on integration of pixel intensities associated with globule compared to background fluorescence.

One method to directly observe protein binding to a nucleic acid (NA) substrate is through imaging the colocalization of fluorescently labeled proteins with the substrate. A previous study utilized thin microchannels (100 by 150 nm) to isolate, straighten, and image single long DNA substrates in the presence of NC protein [23]. Imaging of both the DNA sparsely labeled with an intercalator dye and NC with a covalently-conjugated dye showed the formation of DNA/protein globules along the substrate that were associated with shortening of the DNA’s end-to-end length. We extended this experimental design by using the capability of optical tweezers to manipulate a single long DNA molecule. A single lambda DNA molecule (48.5 kbp), biotinylated at both ends, was tethered between two beads, both held by a movable laser trap (Figure 2A). While the beads display weak autofluorescence, the DNA is unlabeled and does not appear under confocal imaging. However, upon incubating the DNA with 1 μM NC labeled with Alexa 488, a thin line of fluorescent signal is visible directly between the two beads, exactly where the stretched DNA is located (Figure 2B, beads also appear brighter due to nonspecific binding to fluorescently-labeled protein. See also Figure S2 for control experiments with unlabeled NC). This NC concentration is ~2 orders of magnitude higher than required for DNA condensation (determined in the following experiments using unlabeled protein), ensuring complete saturation of the DNA substrate. When the two tethering beads were moved closer together, reducing the extension of the DNA, a bright fluorescent globule appeared along the substrate. Furthermore, continuing to decrease the substrate extension resulted in an even brighter globule. Upon returning the extension to its original length, the increase in fluorescence intensity was reversed (Figure 2C–F). Thus, our results confirm that the number of fluorescently-bound NC proteins incorporated into the globule increases as the DNA becomes more compact, consistent with observations made of individual DNA substrates isolated in microchannels [23]. These results further support the conclusion that NC compacts DNA by incorporating increasing lengths of the DNA as the globule grows.

### 3.2. Force-Extension Measurements Determine the Conformation of DNA-NC Complexes

**Figure 3:**
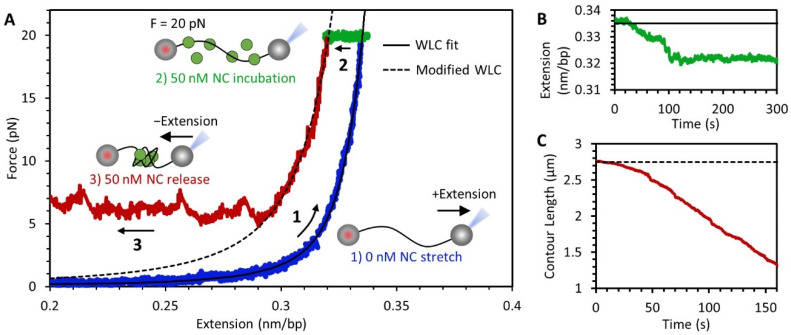
NC-mediated compaction of DNA. (**A**) DNA force-extension curve showing results of an experiment performed in three steps. (1, blue) An 8.1 kbp DNA, biotinylated on both ends, is tethered between two streptavidin coated beads. One bead is held in a stationary dual-beam optical trap while the other is moved using a piezo-electric stage attached to a micropipette tip in order to extend the DNA. Stretching of protein-free DNA follows the idealized polymer WLC model (black line fit to blue data). (2, green) While the DNA is held at a constant tension of 20 pN, a fixed concentration of NC is flowed into the sample and incubated with the DNA. Binding of the NC to the substrate results in a small decrease in extension. (3, red) The extension of the DNA is reduced, initially resulting in a decrease in tension, consistent with the WLC model with modified contour and persistence length (dashed line). Once a critical force is reached, however, the DNA continues to compact without a drop in tension, resulting in a force plateau. (**B**) Plot of DNA extension during protein incubation while holding at a constant 20 pN (green data and black fit line from panel (**A**)). (**C**) Contour length of uncondensed DNA during NC mediated compaction including force plateau (red data and dashed fit line from panel (**A**)).

The conformation of a single DNA molecule over time can be accurately measured using an optical tweezers instrument, which allows for continuous monitoring of the substrate’s extension as force is applied. One advantage to measuring the state of the NC-DNA complex solely through force spectroscopy is that labeling of the protein is not required. A single 8.1 kbp DNA was isolated using an optical tweezers system and a three-step experiment was performed (Figure 3A). First, the DNA was stretched until a constant tension of 20 pN was achieved (Figure 3A, blue line). Before the introduction of protein, the DNA acts as an ideal flexible polymer described by the extensible worm like chain (WLC) model (Figure 3A, black line), with a contour length of *L* = 0.34 nm/bp, persistence length *p* = 45 nm, and elastic stretch modulus *s* = 1200 pN, in agreement with previous measurements [33]. Thus, the protein-free DNA extension (*x*) as a function of applied force (*f*), is well described by:

(1) $$x(f)=L\left(1-\frac{1}{2}{\left(\frac{k_{B}T}{p\cdot f}\right)}^{1/2}+\frac{f}{s}\right)$$

The agreement between experimental data and the model indicates that while increasing the applied force on the DNA substrate increases its extension according to Equation (1), the contour length and persistence length, fundamental polymer properties of the DNA, remain unchanged. Second, while the DNA was held at a fixed tension of 20 pN, a fixed concentration of NC in buffer was flowed into the sample. We observed a small reduction in the DNA’s extension (~1.5% of its protein-free length), which was maintained once the DNA equilibrates with the protein solution (Figure 3A, green). Since the applied force is unchanged, the drop in extension must be caused by changes to the substrate conformation or its fundamental properties. Third, once the DNA-NC complex has equilibrated, the DNA extension was slowly reduced (Figure 3A, red). The applied tension on the DNA initially decreased in a manner consistent with a modified WLC model with smaller contour and persistence length values (Figure 3A, black dotted line). Once the applied force dropped below a certain threshold (5–10 pN), any subsequent reduction in DNA extension did not relax DNA tension. Instead, a force-plateau formed, where the extension of the DNA continues to decrease but tension is maintained. This is not consistent with a polymer with fixed properties. Instead, the DNA-NC complex must be continuously taking up slack generated by moving the DNA ends together, maintaining substrate tension.

The kinetics of DNA compaction during this experiment were resolved by plotting the extension of the DNA over time. After the initial stretch to 20 pN in the absence of protein, the DNA had an extension of 0.335 nm/bp as predicted by the WLC model (Equation (1)). Once protein entered the sample, the extension of the DNA exhibited a small decrease, then maintained a stable value as the DNA substrate equilibrated with the protein solution (Figure 3B). To quantify the compaction of the DNA after protein incubation, we calculated the effective DNA contour length by entering the measured values of DNA force and extension into Equation (1) and solving for L [34]. This value represents the length of DNA that contributes to the extension of the substrate as opposed to the DNA incorporated into a DNA condensate. As the DNA extension was decreased, the contour length was initially 2.75 μm (0.34 nm/bp multiplied by our 8.1 kbp construct length), and the drop in force is consistent with the relaxation of an idealized polymer (Figure 3C). Once the force on the DNA decreased sufficiently to reach the force plateau, the contour length continually decreased, with the removed length being incorporated into a NC mediated condensate. The observed rate of contour length decrease was limited by the rate at which slack in the DNA is generated, which in turn was determined by the rate at which we decreased DNA extension. We found the same force plateau consistently appeared, regardless of this rate (Figure S3), with NC able to quickly compact the DNA substrate as fast as slack is generated, up to 1000 nm/s.

This experiment effectively separates the NC-DNA interaction into two distinct steps. In the next two sections we detail, first, the binding of NC to DNA held at 20 pN (which allows binding but prevents further compaction) and, second, the compaction of NC-saturated DNA as tension is decreased.

### 3.3. NC Binds DNA Straightened under Force through a Simple Bimolecular Mechanism

**Figure 4:**
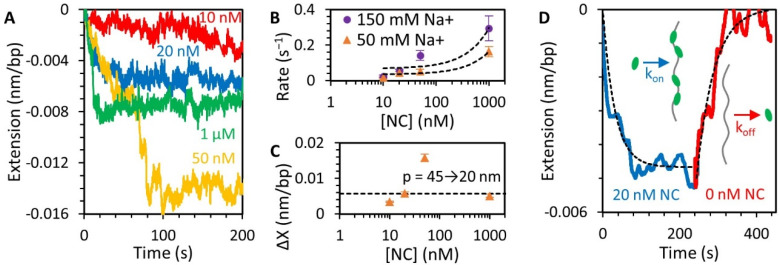
NC binding to DNA above force plateau (Figure 3, step 2). (**A**) Incubation of NC with DNA held at 20 pN results in a small concentration-dependent reduction in DNA extension. Low concentrations of NC (10 nM) result in slow and incomplete shortening of the DNA. At higher concentrations, a faster and larger extension reduction occurs, sometimes occurring in multiple steps before equilibration. (**B**) Binding rates (i.e., the rates at which the NC-DNA complex reaches equilibrium equal to the sum of the rates of free protein binding and bound protein dissociating) increase with [NC] in both buffers containing 50 mM (orange) and 150 mM (purple) Na^+^. The concentration dependence, as determined by a linear fit of the data (dashed lines), is consistent with a bimolecular binding rate of ~10^5^ s^−1^M^−1^. The fits also have a y-intercept of ~0.05 s^−1^, which would imply a fundamental dissociation time constant on the order of 10s of seconds. (**C**) Total change in extension varies with [NC]. Due to multistep compaction, the total extension change observed is largest at 50 nM. If the extension change is primarily attributed to a decrease in DNA persistence length, then the decrease in extension of the DNA at 20 pN in saturating NC (1 μM) is consistent with a halving of persistence length from 45 to 20 nm. (**D**) Dissociation of NC from the DNA substrate is observed when free protein is removed from the sample after incubation. The drop in DNA extension associated with NC incubation is reversed, and DNA returns to its original length on a timescale of ~50 s, consistent with the implied dissociation rate from the y-intercept of the fits in panel (**B**). The observed kinetics are consistent with a simple bimolecular on-off binding mechanism (dotted line fit).

DNA was next held at a fixed tension of 20 pN and incubated with varying concentrations of NC (Figure 4A). DNA shortened over time upon exposure to free NC in solution, with extension exhibiting an exponential drop followed by a steady equilibrium, is as expected for a one-step bimolecular binding process, if we assume that the degree of extension change is directly proportional to the degree of protein saturation along the DNA (i.e., the extension change due to N proteins bound is simply the extension change due to one protein binding multiplied by N). At high concentrations of NC, a secondary drop in extension is often observed after the initial drop. The timing and amplitude of these secondary events varied between experimental replicates. In contrast, at lower concentrations, it takes longer for the DNA and NC to equilibrate, and the net extension reduction of the DNA was reduced (Figure 4B,C). Both these trends (rate proportional to protein concentration and amplitude plateauing at high protein concentration) further support a model of one-step bimolecular binding between the DNA and NC protein, as opposed to the shortening of the DNA requiring a multistep process, such as a subsequent unimolecular conformation change or interprotein interactions between bound NC molecules. The potential biophysical mechanism by which NC reduces the extension of DNA is either through a small decrease in contour length or a large decrease in persistence length. The high applied force of 20 pN would presumably inhibit most contour length reducing structures (DNA loops, protein wrapping, etc.). Since the substrate is nearly straight at 20 pN tension, changes in persistence length only have a minor impact on extension, similar to the ~1.5% change we observe. A previous single molecule fluorescence resonance energy transfer (FRET) assay demonstrated that NC can bend a DNA substrate [35], and these bends would reduce the effective persistence length of the substrate. If we assume the drop in DNA extension is primarily due to a reduction in persistence length, the ~0.006 nm/bp reduction in extension we observe in the presence of saturating NC is consistent with the DNA persistence length being halved from *p* = 45 to 20 nm. This result is physically reasonable as *p* is still longer than the binding site size of NC, and thus could be caused by localized bending of the DNA where NC is bound. We further show that the WLC chain model using a reduced persistence length fits well to a stretching curve of the protein-saturated DNA (Figure 3A, dotted line), though the fit is most sensitive to reductions in *p* at low force where the force plateau prevents direct comparison.

We also tested the reversibility of NC binding by removing all free protein from the sample after the DNA substrate was saturated with NC (Figure 4D). Once free protein was removed, the DNA substrate slowly elongated, approaching its original protein-free length. This is consistent with the bound NC dissociating from the DNA on a 100 s timescale. Thus, our results are consistent with NC binding the straightened DNA substrate at 20 pN as a simple, reversible bimolecular process.

### 3.4. NC Mediates DNA Compaction

**Figure 5:**
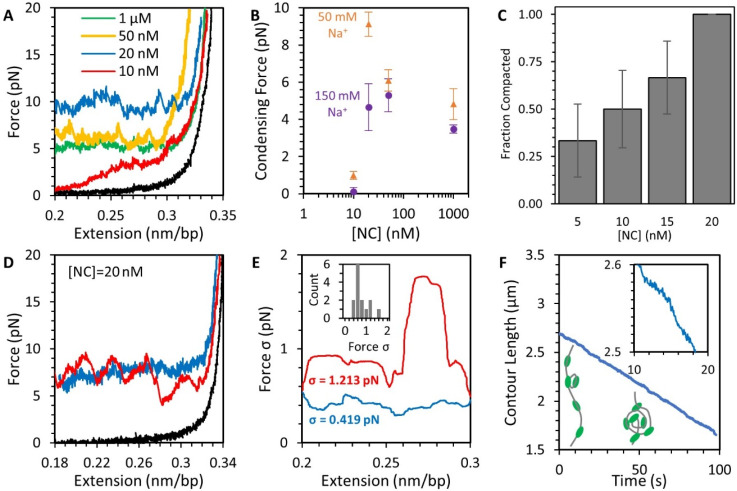
Compaction of DNA by NC at low force (Figure 3, step 3). (**A**) Decreasing the extension of the DNA substrate reduces its applied tension and allows for NC-mediated compaction depending on NC concentration (colored lines, black line shows protein-free DNA for reference). At saturating concentrations of NC (>20 nM), a force plateau emerges, where the NC condensate pulls against the stretching force and absorbs all excess DNA, preventing further drops in tension. At sub-saturating NC concentrations (10 nM), the DNA is not fully compacted and its tension approaches zero. (**B**) The average compaction force as a function of NC and Na^+^ concentration shows a minimum of ~20 nM NC is needed for efficient compaction and a slight decrease in compaction force at higher protein concentration. (**C**) The fraction of DNA substrates that compact at low force (exhibit a force plateau) increases with NC concentration. (**D**) Individual force plateaus may appear rough or smooth as the force fluctuates moderately (blue) or substantially (red) as the DNA compacts. (**E**) The fluctuations in force, as measured by standard deviation of force (σ), are measured along the same force plateaus plotted in panel (**D**) using a moving average of 10 s. The rough plateau (red) shows greater deviations in force along its entire length as compared to the smooth plateau (blue) (average deviation over entire curves are listed on plot). The inset displays a histogram of individual DNA molecules compacted in the presence of NC. (**F**) Using instantaneous force and extension measurements, the contour length of the DNA-NC complex is calculated over time showing increasing DNA condensation as illustrated by inset cartoons. Instead of discrete steps, the length of the polymer decreases gradually as seen in the magnified inset. The instantaneous slope of this line varies over time, especially for DNA compactions with larger force deviations, but the contour length always decreases monotonically over time with an average rate determined by our instrument controls (i.e., the rate at which the beads are moved closer).

Once the extension of the DNA-NC complex is reduced, the tension on the substrate initially decreases as well. When the applied force is lowered to a critical value (F_c_), NC-mediated condensation of the DNA commences, forming a force plateau (Figure 3, red line). Any additional release of the substrate’s extension does not ease its tension, as all generated slack on the polymer chain is concurrently incorporated into the condensed globule. This compaction occurs at a fast rate, as the plateau occurs independent of the rate at which DNA extension is decreased (Figure S3), with even a 1000 nm/s rate of release unable to generate tension relieving slack, indicating the DNA was incorporated into the NC-mediated condensate faster than the ~100 ms response time of our instrument. We observed this compaction and resulting force plateau in the presence of various concentrations of NC in solution (Figure 5A,B). The plateau forms for NC concentrations ≥20 nM, consistent with the observed saturated binding at the same concentration discussed above. At lower concentrations, the DNA will often only partially compact (Figure S4). Moreover, the probability that DNA has at least a partial force plateau increases with NC concentration in the range from 5 to 20 nM (Figure 5C). This partial compaction likely indicates NC is not evenly distributed along the substrate, but instead redistributes along the DNA to saturate and condense only part of its length, similar to observations of finite size globules forming on a DNA complex at sub-saturating concentrations [23].

The shape of the force plateau also contains details concerning the structure of the NC-mediated condensate. If DNA was being looped, folded, or otherwise compacted into a defined, repeatable structure, the force plateau should exhibit a periodic pattern where the substrate alternates between slowly relieving tension and experiencing a sudden force jump as a new compact structure is formed. Such a pattern of discrete steps was observed for DNA toroids formed by spermidine (Spd^3+^) [34]. However, we observed high variability in compaction shape between replicate experiments performed under the same conditions. Different DNA molecules compacting in the presence of 20 nM NC at the same average force of 8 pN (Figure 5D) had either a smooth or rough appearance. The smooth curve is a result of the DNA continuously compacting with no large-scale sudden events occurring, while the rough curve has several interspersed events where the substrate compacts substantially in a short period of time. The roughness of the curve can be quantified using the standard deviation of force along the force plateau (Figure 5E). This feature is not just a function of stochasticity in time, as any DNA that starts compacting with a rough shape will continue to do so for the rest of the experiment. We also inspected the contour length of the uncondensed DNA over time for any evidence of condensate structure (Figure 5F). If the substrate compacts into discrete repeatable structures, such as loops being added to a toroid [34], this data would form a step function, where the amplitude of each step is equal to the length of DNA being incorporated into the structure. Instead, we found that the DNA-NC complex compacts in a continuous fashion, with no steps visibly larger than our instrument resolution (1 nm and 0.1 pN). Indeed, attempting to fit the data to a step function (Figure S5), such as one due to the formation of repeated formation of DNA loops, did not adequately describe this system.

### 3.5. Stability of the NC-DNA Complex

**Figure 6:**
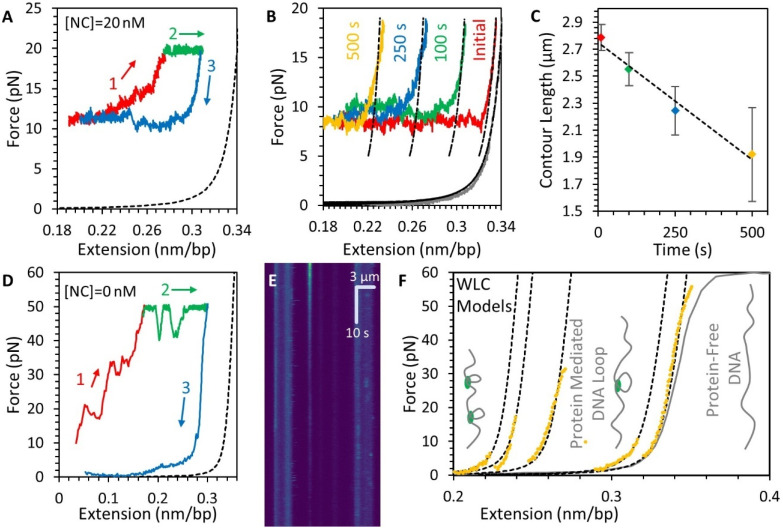
Stability and structure of the DNA-NC complex. (**A**) After the -NC-DNA condensate is formed (as shown in Figure 3, Figure 4 and Figure 5), the complex is re-stretched (1, red), displaying large hysteresis, indicating that NC compaction is not reversible. High force can partially decompact the DNA (2, green) and the force plateau reappears when the extension of the DNA is decreased (3, blue). Dashed line shows protein-free DNA for reference. (**B**) When the compacted DNA substrate is left incubating with NC (20 nM), the NC-DNA complex is progressively shortened, as shown by representative force-extension curves with different incubation times (colored lines). (**C**) The contour length of the NC-DNA complex decreases as incubation time is increased. (**D**) Re-stretching the NC-DNA complex immediately after free protein is removed from the system still requires high applied force for decompaction (1, red and 2, green), but the majority of NC is dissociated from the DNA as the release of the DNA extension no longer displays the force plateau caused by NC-mediated compaction (blue). Dashed line shows protein-free DNA for reference (**E**) Kymograph showing globules of fluorescently-labeled NC initially formed at low DNA tension (top) dissipating while the DNA is held at a tension of 20 pN (same condition as green line in panel (**D**)). Horizontal axis shows fluorescence intensity along axis of stretched DNA with large stripes on left and right displaying bead autofluorescence and bright spots in between indicating the locations of NC globules. Vertical axis shows the globules fading but not disappearing entirely over 10 s timescale, indicating partial dissociation. (**F**) Re-stretching the NC-DNA complex again, still in the absence of free protein, follows WLC models with reduced contour length (dashed lines). Several sudden abrupt increases in contour length (shifting to the right) occur, consistent with the sudden breaking of large DNA loops, until the DNA reaches the extended length of protein free DNA (gray line), as illustrated by inset cartoons.

Since the majority of DNA compaction occurs along the force plateau, forces above the plateau presumably impede compaction. To check if such high forces are capable of also destabilizing the condensate after it forms, we extended the DNA-NC complex after compaction has occurred (Figure 6A). This experiment was performed directly after the experimental procedure detailed in Figure 3, such that the DNA is compacted to approximately half its fully extended length along the force plateau. If the formation of the NC condensate was reversible, the stretching of the DNA would follow the force plateau in reverse until the DNA was fully extended, as was previously seen for toroidal compaction of DNA [34]. Instead, we observed that stretching the DNA-NC complex resulted in an increase in substrate tension, indicating the NC condensate persisted at higher forces. Continuing to hold the DNA at a 20 pN force resulted in partial elongation of the substrate, though some degree of compaction remained. Finally, decreasing the extension of the DNA again resulted in an initial drop in tension followed by the same force plateau seen in the initial compaction. These results show that while the NC condensate quickly forms given sufficient slack in the DNA, the condensate is resistant to disruption via force. This is further shown by repeating the stretching experiment after allowing the DNA-NC complex to incubate at low extension for an extended period (Figure 6B,C). The longer the NC condensate is allowed to form, the shorter the remaining uncondensed DNA, as measured by the contour length of uncondensed DNA upon stretching to a tension of 20 pN.

We confirmed that high force partially decompacts the DNA-NC complex by repeating this stretching experiment after removing free protein from solution (Figure 6D). The DNA-NC complex was held at a force of 50 pN for 2 min while continuously flowing protein-free buffer into the sample, so that dissociated NC would be removed from the sample. Again, the substrate partially elongated, displaying both slow increases in extension over time and sudden rips that rapidly increase length, but the DNA did not reach its full extension. Without free protein in solution, decreasing the DNA extension allows for the full relaxation of the substrate, without a force plateau. We made a similar observation using confocal imaging of the globules formed by fluorescent protein. After discrete labeled NC globules form on the substrate at low extension (as shown in Figure 2), the DNA’s tension is increased to 20 pN. A kymograph shows that these globules dim, but do not entirely disappear, on the timescale of 10s of seconds (Figure 6E). The fluorescence dimming is not primarily driven by photobleaching, as this decrease in fluorescence intensity was also observed in sequential discrete confocal images where the sample is not being continuously exposed to the excitation laser, and the fluorescence intensity of the globules is stable at low, non-destabilizing force (Figure 2). These images compare favorably to the force extension data for the same conditions (Figure 6D green), which show partial dissolution of the NC-mediated condensate under tension.

Finally, stretching the now mostly protein-free DNA strand shows a series of extension jumps as force is increased (Figure 6F). The force-extension curve of the mostly protein-free DNA shows that the substrate acts like an idealized WLC, but with reduced contour length. The sudden force jumps indicate near instantaneous increase in contour length until the full length of protein free DNA is achieved. This result is consistent with a small amount of non-dissociating NC stabilizing large loops of otherwise protein-free DNA that are pulled apart at higher force.

### 3.6. NC Directly Compacts DNA at Low Force

**Figure 7:**
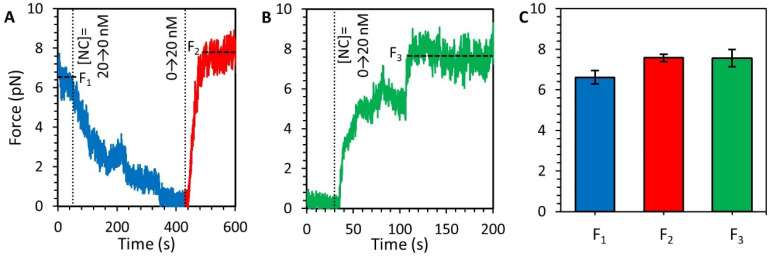
(De)compaction of DNA-NC at low force. (**A**) A DNA that is saturated and compacted by 20 nM NC is held at an extension of roughly half that of fully extended DNA, following the experimental procedure outlined in Figure 3. Free protein is removed from the sample (blue) and over hundreds of seconds the tension on the DNA decreases as the DNA decompacts. Reintroducing the NC results in immediate recompaction of the DNA (red). Vertical dotted lines indicate changing NC concentration and horizontal dashed lines indicate equilibrium extension. (**B**) Protein-free DNA is held at a fixed extension of approximately half its fully extended length, resulting in low substrate tension (<1 pN). NC is flowed into the sample and incubated with the DNA, resulting in an increase in substrate tension over time. The force on the DNA eventually plateaus at the same value observed in previous experiments. (**C**) Comparison of compaction force for different experimental procedures show that the force plateau generated by NC-mediated compaction of DNA is consistent for the first compaction after extension reduction (F1, blue), the second compaction after reintroduction of NC into the sample (F2, red), and spontaneous compaction upon introduction of NC to protein-free DNA (F3, green).

The experimental procedures detailed above separate NC binding and compaction into two distinct steps. However, in the virion viral DNA is constrained by the capsid into a tight volume, rather than extended into the straight conformation in our experiments. Thus, inside the viral capsid, it is possible for NC to bind newly polymerized DNA and incorporate the growing strand into the compacted structure in one combined step. We replicated such a scenario by holding the DNA substrate at approximately half its extended length while introducing and removing the protein such that we observe binding-compaction and dissociation-decompaction each in one combined step. We started with compacted DNA (Figure 3). While keeping the DNA extension fixed, free protein was removed from the sample by continuous flow of protein-free buffer (Figure 7A). Over the timescale of 10 min, the tension on the substrate dropped and approached zero. This indicated enough NC had dissociated from the DNA to allow for enough decompaction to provide adequate slack along the substrate. NC (20 nM) was then flowed back into the sample, causing the tension on the DNA to increase back to the original force plateau, indicating the free NC binds and compacts the DNA. We see the same results if we start with a protein-free DNA that was never introduced to NC (Figure 7B). In this case, we held the DNA at a fixed extension of approximately half its fully extended length, such that the DNA is under minimum tension (<<1 pN). A fixed concentration of NC was then flowed into the sample chamber. The tension on the strand increased over time, reaching an asymptote (Figure 7B). The maximal compaction force achieved due to NC-mediated compaction was conserved in these different experimental procedures (Figure 7C). Thus, the compacting force generated by NC is an intrinsic equilibrium value indicative of the strength of the NC-mediated condensate.

### 3.7. AFM Imaging Reveals the Size and Shape of Condensed DNA-NC Complexes

**Figure 8:**
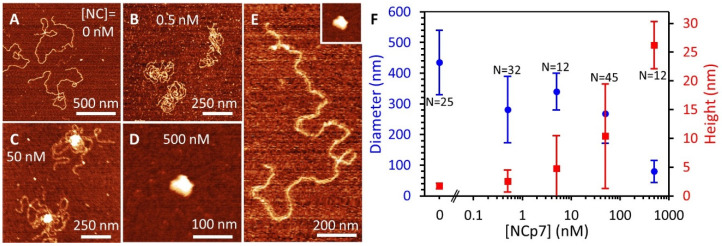
AFM images of NC-DNA complexes. (**A**) A 7.2 kbp DNA molecule, in the absence of protein, assumes a spread-out conformation, consistent with the WLC model, with infrequent DNA overlaps. (**B**) Low concentrations of NC (<10 nM) stabilize crosslinking of DNA loops, reducing the spread of the DNA but not producing a condensed globule. (**C**) Moderate NC concentrations create a highly condensed DNA globule that partially condenses the DNA. (**D**) Saturating quantities of NC (>100 nM) condense the entire DNA molecule with no bare DNA remaining. Note the panels show different length scales. (**E**) Comparison of a protein-free DNA and fully compacted DNA-NC globule (inset) on the same scale. (**F**) Average diameter and height of DNA molecules as a function of NC concentration, with total number of DNA molecules measured (N) listed for each condition. Increasing NC compacts the DNA, resulting in a smaller maximum DNA diameter on the surface (blue) and higher maximum height due to the formation of a tight 3D globule consisting of many layers of DNA (red). Error bars represent standard deviation, showing the spread of values for individual DNA molecules.

As an alternative method of determining the compacting capabilities of NC, we carried out AFM imaging of long DNA (7.2 kbp) in the presence of NC. DNA molecules were incubated with increasing concentrations of NC, deposited on a nickel-treated mica surface that attracts and captures the DNA, and imaged using a sharp AFM tip (~2 nm). Preliminary experiments using higher concentrations of DNA (~1 nM to ensure coverage of the mica surface) and longer incubation times (~5 min to ensure full binding of NC) showed extensive intermolecular aggregation of DNA molecules in the presence of NC. That is, multiple 7.2 kbp DNA molecules were conjoined into one structure, which was apparent both by visual inspection and volumetric analysis of the aggregates. To minimize intermolecular DNA aggregation, the DNA sample was diluted to 50 pM before the introduction of NC (less than one DNA molecule per μm^2^ of mica surface), and incubation was limited to one minute before deposition on the surface, allowing us to observe and measure single DNA molecules. The AFM images provide a direct measurement of the conformation of the DNA-NC complex (Figure 8A–E). While protein-free DNA acts like a polymer chain confined to a 2D plane (Figure 8A), NC compacts the substrate, first by stabilizing crosslinking of the DNA to form loops (Figure 8B), and then through the formation of a central globule structure (Figure 8C) that eventually compacts the entire DNA molecule under saturating NC (Figure 8D). We quantified this process by measuring the average diameter (distance from leftmost to rightmost and topmost to bottommost edge) and maximum height of every imaged DNA molecule. Plots of the average value along with standard deviation for every condition are shown in Figure 8F. For protein-free DNA, the molecule is spread out over an average of ~450 nm (with large deviations due to the inherent stochasticity of a single molecule conformation) and a maximum (and uniform) height of ~1.5 nm (the effective diameter of bare DNA). As NC was added, we observed a small decrease in diameter due to increased loop formation (Figure 8B), which also resulted in a slightly increased maximum height as the DNA crosses over itself. The maximum height increased around 10 nM NC as the central globule started to form (Figure 8C). Since the total amount of the DNA molecule incorporated into the globule is variable, the size of the globule also shows large variability. Once saturating quantities of NC are added (>100 nM), the entire DNA molecule is incorporated, resulting in a more uniform large compact globule (Figure 8D). Due to the attraction between the sample and the surface, required for AFM imaging, the globule is slightly deformed against the surface and slightly wider (diameter) than it is tall (height). By measuring the total size of the globule through pixel integration, we found that the fully condensed globules to average 23,000 ± 8000 nm^3^ in volume. For comparison, under conditions that allowed for intermolecular DNA aggregation (higher DNA concentration and longer incubation time), we observed much larger volume structures on the surface with a wide distribution of values, indicating DNA multimerization, emphasizing the importance of dilute samples to ensure single DNA molecules are observed. We can estimate the volume of the NC-coated DNA prior to condensation as a long cylinder with length 2.45 μm (7200 bp⋯0.34 nm/bp). Considering the DNA by itself has an effective diameter of ~2 nm, the bound protein would result in an effective diameter of ~3 nm or greater. Thus, comparing these two values, the uncondensed and condensed forms of the DNA-NC complex have the same approximate volume, indicating that NC mediated condensation compacts the DNA as much as is sterically possible, and that the globule does not have a central hole or other hollow features.

## 4. Discussion

### 4.1. NC-Induced DNA Condensation Is Similar to That of Other Multivalent Cations

In this study, we observed that at saturated NC binding, DNA was compacted into a maximum density globule, reaching a minimum volume limited by steric interaction. DNA condensation by NC qualitatively resembles DNA condensation induced by many other multivalent cations with an effective charge Z ≥ +3 [25,36,37]. We compare our results from this study with a summary of previous works in Table 1. NC has an effective charge of only +3.5 [38,39], despite the presence of 15 cationic and only 4 anionic residues. The effective charge is defined as the number of Na^+^ (or other monovalent cations) released into bulk solution upon protein/nucleic acid binding [40,41,42,43]. Only the cationic residues that come into close contact with the nucleic acid phosphates upon binding contribute to its effective charge [42,44]. The physical mechanism of NC-induced DNA condensation is likely electrostatic counterion correlation [45,46,47], as suggested by several features of the NC-induced DNA condensation observed in this work. However, alternative mechanisms of DNA condensation by multivalent counterions originating from the patterns of cationic absorption on the helical DNA surface have been suggested [48,49]. The condensation force plateau appears above a critical minimum concentration of condensing cation (c_min_) which increases with salt, as condensation requires DNA saturation with multivalent cations, while its primarily electrostatic DNA binding is strongly reduced by salt [25,37]. Moreover, multivalent cation-induced DNA condensation forces have been observed to reach a maximum at an intermediate concentration (c_mid_) and then disappear at a higher concentration (c_max_) as DNA reverses its effective charge and re-solubilizes back into solution [50,51] (see comparison in Table 1). Such re-entrant DNA condensation was theoretically predicted from the counterion-correlation mechanism of DNA condensation [47] and was observed in multiple bulk solution studies in the same range of condensing cation concentrations that also lead to DNA charge neutralization and reversal [51,52]. We find that the NC-induced condensing force plateau appears at [NC] > 10 nM (in 50 mM Na^+^) and increases with NC concentration to a maximum value of nearly 10 pN at ~20 nM NC. Further increases in NC concentration lead to a decreasing condensing force, down to ~5 pN at 1 μM NC (Figure 5B), consistent with the expectations based on models for DNA condensation discussed above. Strong NC-induced DNA self-attraction (as compared to analogous DNA self-attraction caused by other similarly charged cations, Table 1) likely results from significantly stronger NC-DNA binding and slower dissociation due to additional non-electrostatic contributions to binding. Slow NC dissociation kinetics could also lead to more pronounced NC charge periodicity on each DNA surface and an accompanying stronger attraction between such DNA surfaces with complementary charge patterns [45].

### 4.2. NC Exhibits Exceptionally Strong DNA Binding Compared to Other DNA Condensing Ligands

The critical NC concentration of ~10 nM required for DNA condensation is in agreement with the DNA dissociation constant K_d_ ~10 nM previously measured by DNA-NC complex stretching [55], and is significantly below critical condensing concentrations of other tri- and tetra-valent cations cobalt hexamine (CoHex^3+^), Spd^3+^, and spermine (Spe^4+^) (Table 1). NC/DNA binding is thus ~4 orders of magnitude stronger than simple trivalent cations CoHex^3+^ or Spd^3+^ with typical K_d_ values of 10–100 μM [51,54]. This enhancement of NC/DNA binding implies an increased binding free energy of ~ln(10^4^) = ~10 k_B_T per protein. This enhancement likely results from non-electrostatic interactions, primarily from the stacking of the two aromatic residues located in the zinc knuckle domains of NC with unpaired bases. Indeed, the DNA intercalative propensity of NC is known to be promoted by the stretching of DNA to forces above 40 pN [56,57,58,59]. The strong non-electrostatic binding of NC was also observed in a salt-titration assay, when the electrostatic contribution to binding was salted out by addition of 1 M NaCl [39].

### 4.3. NC Lowers the DNA Persistence Length

A large non-electrostatic component to NC/DNA binding is consistent with the reduction in DNA persistence length observed in this work, as well as with previous single-molecule FRET measurements [35]. The NC-induced persistence length reduction far exceeds the analogous effect of other multivalent cations and the predictions for the electrostatic effects of multivalent cations on DNA flexibility [53,60,61]. It is likely that NC’s ability to transiently and partially intercalate DNA significantly increases dynamic DNA flexibility, as was previously observed for other intercalating proteins like High Mobility Group (HMG) proteins [62,63,64].

### 4.4. NC Induces Strong DNA Self-Attraction

NC induces an exceptionally strong DNA condensation force F_c_ = 9 ± 1 pN, and its corresponding DNA self-attraction free energy per base pair (G_DNA_ ≈ 9 pN⋯0.34 nm/bp = 0.8 k_B_T per bp) is significantly larger than that observed for any other multivalent cation previously studied. Such strong self-attraction, in combination with the NC-induced decrease in DNA persistence length, enable NC to compact DNA into a space-filling dense globule. This is in contrast to prior observations with multivalent cations, in which DNA condenses into a toroidal globule with a measurable large inner hole and DNA strands winding parallel to each other. This maximizes DNA side-by-side self-attraction and minimizes its bending. Theory predicts a critical DNA length (*L_cr_*) above which the DNA globule transitions from its toroidal conformation to a densely packed globule with no central hole [21]:

(2) $$L_{cr}\approx \frac{1}{d^{5}}{\left(\frac{b\cdot p}{\alpha }\right)}^{3}$$

Based on measured values of length per B DNA base pair (*b* = 0.34 nm), persistence length (*p* = 20 nm), B DNA diameter (*d* = 3.2 nm), and DNA self-attraction free energy per bp (*α* = 0.8 k_B_T), this equation predicts that above a critical DNA length (*L_cr_* = 1 nm) NC will form a dense globule rather than a toroid. Thus, the DNA-NC complex is predicted to always form a dense globule, a hypothesis that is supported by two independent experiments. First, the NC-induced condensation force plateau does not show a regular sawtooth structure with periodicity of 30–50 nm as described for Spe^4+^, which would signify DNA winding into a toroid [34]. Force fluctuations observed around the condensation plateau with NC are typically much smaller and with poorly defined periodicity, suggesting curvature of the globule that is below our resolution limit. Secondly, the volume of DNA globule observed by AFM for DNA maximally condensed by NC is similar to the self-volume of DNA of the given length. The effective diameter of 3.2 nm is consistent with a DNA diameter of 2 nm plus a 1.2 nm layer of NC proteins. This also provides an estimate of the net volume of the DNA globule as DNA is synthesized during reverse transcription inside the capsid, which is important when considering the mechanism of reverse transcription-induced capsid uncoating.

### 4.5. DNA Condensation by Sub-Saturating Amounts of NC Leads to Phase Separation

DNA condensation by multivalent cations is known to proceed as a highly cooperative transition, with the whole DNA collapsing into its globular state at concentrations of condensing agent above a critical value [25,37,65]. This cooperative first-order phase transition was observed for all multivalent cations that condense DNA through a variety of experimental techniques, including single molecule DNA force-extension studies, which observed the entire DNA molecule compacting at a well-defined condensation force into a globule [34,50,51,54]. However, in this work, we observe that sub-saturating amounts of NC (<20 nM) lead to only a part of the DNA molecule condensing into a globule. This result suggests that NC-induced DNA condensation is able to phase-separate sub-saturating amounts of NC from being uniformly spread along the whole DNA, instead condensing it into an NC-saturated condensed DNA region alternating with an NC-free uncondensed DNA region. We hypothesize that this is a consequence of the extremely strong NC-induced DNA condensation, which drives NC phase separation along the DNA molecule. Similar phase separation into an NC-saturated condensed and NC-free uncondensed DNA molecule was recently observed for fluorescently-labeled NC bound to YOYO-1 labeled DNA constrained to a microchannel [23]. This study also correlated the size of the condensed globule with the length of remaining uncondensed DNA, showing that as the NC globule grew, the length of uncondensed DNA shrank a proportional amount. We observed this feature in our confocal imaging of labeled NC in our optical tweezers experiments, where DNA extension is measured using the position of the tethering beads rather than by binding DNA with intercalating dye (Figure 2F). We also observed this feature in our AFM data (Figure 8), where the DNA begins to form a globule with some DNA remaining uncondensed at moderate NC concentrations (Figure 8C).

### 4.6. Two Distinct Modes of NC/DNA Interaction and Compaction Are Likely Critical for Viral Capsid Uncoating

We observe NC-DNA binding over a wide range of conditions, identifying two major modes of NC-induced DNA compaction and their associated kinetics. We find that the majority NC associates with DNA relatively fast with a bi-molecular rate k_on_ ~ 10^5^ M^−1^s^−1^ and dissociates with an average rate k_off_ ~ 10^−2^ s^−1^ (Figure 4). This binding mode promotes condensation into a globule along the condensation force plateau, as adding or removing NC from either the stretched or relaxed NC/DNA complex leads to the concurrent appearance or disappearance of the DNA condensation force plateau (Figure 7). However, even when NC is washed from the substrate for up to ~1000 s with no remaining condensation force plateau, the presence of a small amount of long-lived NC bound to DNA is still evident in the DNA stretching curve (Figure 6E). This DNA stretching pattern suggests long-lived NC molecules that crosslink large (>1 μm) DNA loops and are only destabilized by a large applied force. Similar patterns of NC-induced DNA looping into an only slightly compacted structure were observed in our AFM images at very low NC/DNA saturation levels (Figure 8B). This mode of NC-DNA binding and condensation operates at very low NC/DNA saturation, when NC binds only to specific sites on DNA with high affinity and slow dissociation kinetics. This binding mode likely relies on NC-DNA interactions beyond simple electrostatic interactions, possibly including local stacking of the aromatic residues of NC with unpaired DNA bases, either in a duplex-intercalative mode, or by local DNA bp extrusion. Different NC-nucleic acid (NA) binding modes were previously documented [35,66,67], with the majority of NC molecules binding nucleic acids in a salt-dependent manner while retaining high mobility and relatively fast dissociation [68], and a minor population binding to a few specific sites via non-electrostatic interactions [66,67].

This range of NC-DNA dissociation kinetics may play a major role during reverse transcription, when proviral DNA synthesis doubles the total amount of polymerized nucleic acid inside the capsid, while the total amount of NC remains constant. Thus, the progress of reverse transcription is expected to lead to sub-saturating amounts of NC within the capsid, which is insufficient to condense the complete newly-synthesized proviral DNA. Very strong NC-induced DNA condensation observed in this work may imply that NC re-distributes from viral RNA to DNA to condense some fraction, even under sub-saturating conditions. Uncondensed NC-free DNA will form large DNA loops that are supported by specifically-bound NC molecules, gradually leading to a buildup of pressure inside the capsid, eventually leading to capsid uncoating [69]. As the kinetics of NC dissociation and DNA de-compaction is slow, relative to other multivalent cations, this NC re-distribution can take on the order of hours, consistent with the hour-long lag time between the completion of reverse transcription and capsid uncoating in the nucleus [3,4,5,6,10]. Once the capsid integrity is initially compromised by the first rupture in its structure, the electrostatically-bound majority of NC proteins will quickly (within 10–100 s) dissociate into the cell, leading to complete DNA de-condensation and concurrent capsid disintegration. This hypothesis regarding the relationship between the DNA-condensing role of NC, the kinetics of NC dissociation from DNA, and capsid uncoating will require further experiments both in vitro and in infected cells. The role of other DNA binding proteins, such as IN, which may affect NC condensation, should also be investigated [70,71].

## Figures and Tables

**Table 1:** Comparison of DNA condensation properties of NC and other multivalent cations. Summary includes buffer conditions of experiments, cation concentrations resulting in the onset, maximization, and end of DNA condensation (C_min_, C_mid_, C_max_), maximum force plateau (F_max_), maximum free energy associated with DNA condensation per bp (G_DNA_), persistence length of DNA saturated with cation (p_min_), the degree of hysteresis between stretching and release of DNA condensate, and the measured timescale of DNA binding.

Multivalent Cation [Reference]	Buffer	C_min_ C_mid_ C_max_	F_max_ (pN)	G_DNA_ (k_B_T)	p_min_ (nm)	Hysteresis during Stretching	DNA Binding Kinetics
Spd^3+^ [53,54]	10 mM NaHPO_4_, pH 7	200 μM N/A N/A	1	0.08 ± 0.01	37	Large	
CoHex^3+^ [53,54]	10 mM NaHPO_4_, pH 7	25 μM N/A N/A	4	0.32 ± 0.05	18	Large	
Spd^3+^ [50]	10 mM Tris-HCl, pH 7	0.5 mM 2 mM 100 mM	1.8 ± 0.2	0.15 ± 0.05		Grows with [Spd^3+^] and condensate size	
Spe^4+^ [34]	10 mM Tris 1 mM EDTA, pH 7		4.5		35	Moderate after condensate formation	Second timescale
Spe^4+^ [51]	10 mM Tris 50 mM NaCl	10 μM 10 mM 1 M	2.2			Moderate after condensate formation	Second timescale
NC^3.5+^ [This Work]	50 NaCl 10 mM Hepes, pH 7.5	10nM 20nM >1 μM	9 ± 1	0.85 ± 0.1	20	Large, increases with incubation	10–100 s timescale

## Data Availability

The data presented in this study are available on request from the corresponding author.

## Supplementary Materials

The following are available online at https://www.mdpi.com/article/10.3390/v14020235/s1, Table S1: Oligonucleotides used in synthesis of biotinylated DNA constructs, Figure S1: Fluorescent labeling of A54C NC protein, Figure S2: Fluorescence imaging control with unlabeled NC, Figure S3: DNA compaction as a function of rate of extension change, Figure S4: Variability of low NC concentration compaction, Figure S5: Step fitting of compaction data.
