# Supplementary material for: HIV-1 Nucleocapsid Protein Binds Double-Stranded DNA in Multiple Modes to Regulate Compaction and Capsid Uncoating
Source: Viruses. 2022 Jan 25;14(2):235. doi: 10.3390/v14020235 (PMC8879225; doi:10.3390/v14020235)
Supplement: Supplementary file 1 [file viruses-14-00235-s001.zip › viruses-1532565-supplementary.pdf]

viruses

MDPI

Supplementary Table and Figures: HIV-1 Nucleocapsid Protein Binds Double-Stranded DNA in Multiple Modes to Regulate Compaction and Capsid Uncoating

Helena Gien, Michael Morse, Micah J. McCauley, Jonathan P. Kitzrow, Karin Musier-Forsyth, Robert J. Gorelick, Ioulia Rouzina, and Mark C. Williams

**Biotinylated Lambda**	**Sequence (5′-3′)**
5′ Biotin	TTT TTT TTT TTT AGA GTA CTG TAC GAT CTA GCA TCA ATC TTG TCC
Linker	GGG CGG CGA CCT GGA CAA
3′ Biotin	AGG TCG CCG CCC TTT TTT TTT TTT
**Biotinylated pBACgus11**	**Sequence (5′-3′)**
5′ Biotin	CTC TCT CTC TCT TCT CTC TTC TCT TGG CCA GCT
Linker	GGC CAA GA
3′ Biotin	GAT CTC TCT CTC TCT CTC TC
Linker	GAG AGA GA

Table S1: Oligonucleotides used in synthesis of biotinylated DNA constructs. Red labeled nucleotides contain an attached biotin.

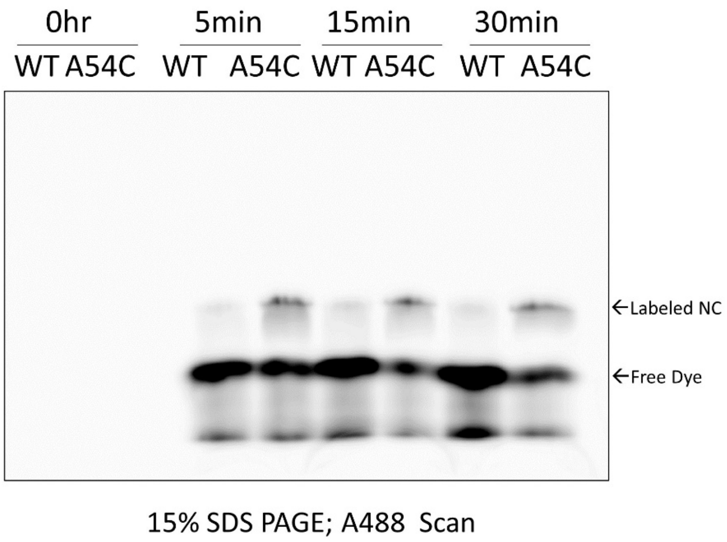

Figure S1: Fluorescent labeling of A54C NC protein. Denaturing polyacrylamide gel comparing Alexa 488-labeled NC gel band for A54C vs WT NC. The majority of bound Alexa 488 dye is specifically attached to the additional Cys residue at position 54. Based on quantification of the bands, we estimate ~5% off-target labeling of Cys residues that are part of the two zinc fingers of NC.

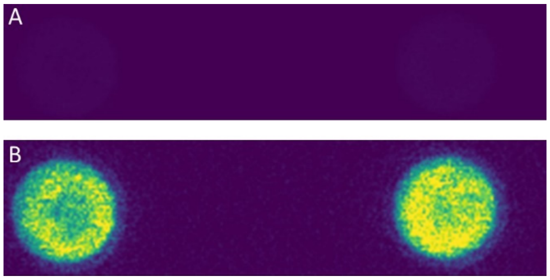

Figure S2: Fluorescence imaging control with unlabeled NC. Confocal images were taken of a DNA substrate tethered between two beads and held at a force of 20 pN in the presence of 1 μM concentration of unlabeled WT NC. Only weak autofluorescence of the beads is observed with no significant background fluorescence in the buffer or localized to the DNA strand. Same image is shown with normal contrast (A, comparable to Fig. 2A) and 40X enhanced contrast (B, comparable to Fig. 2B).

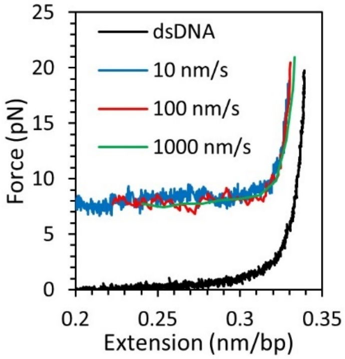

Figure S3: DNA compaction as a function of rate of extension change. At all rates tested over two orders of magnitude, the force plateau caused by NC mediated compaction occurs independent of the DNA's rate of extension change. Note, the data from the faster curves appear smoother simply due to fewer data points being taken over the timescale of the experiment.

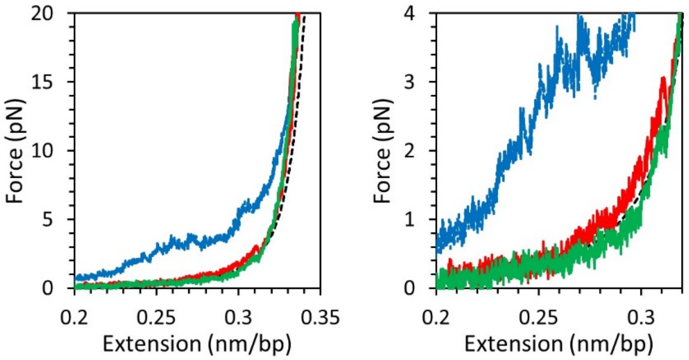

Figure S4: Variability of low NC concentration compaction. At NC concentrations slightly below saturation (10 nM shown here), the degree to which DNA compacts is variable. The substrate often starts compacting at low force but is not saturated enough with NC to fully compact, resulting in the applied force eventually decaying to zero. Three replicate experiments are plotted, showing moderate compaction (blue), a single compaction event (red), and no compaction (green).

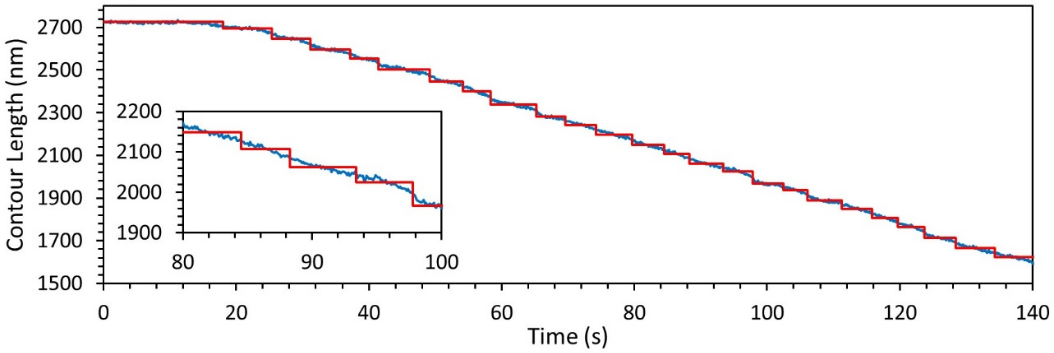

Figure S5: Step fitting of compaction data. Force-extension data for DNA with slowly decreased extension (1 nm steps at ~10 Hz) in the presence of 20 nM NC is converted to a contour length over time (blue line). The contour length decreases continuously, without any discrete steps larger than the noise detection limit of our instrument. For comparison, a best fit step function is plotted (red line) with an average step size of 50 nm, as predicated if the DNA-NC complex compacted by forming discrete loops of DNA. The inset shows a magnified view, with the contour length decreasing in a continuous manner.
